# Medical Items

**Published:** 1897-08

**Authors:** 


### MEDICAL ITEMS.

We are requested to announce that there is a good country practice in Southwest Georgia free to any young physician. Address H. Wilson, M.D., Doles, Ga.

Drs. W. S. Elkin and H. P. Cooper announce that their sanatorium in Atlanta is rapidly approaching completion and that it will be ready for patients about October 1st. The building will be a handsome structure of brick and stone, four stories high, with fifty-two rooms. It will be thoroughly modern and provided with all conveniences and apparatus for a well appointed surgical sanatorium.

Dr. A. B. Calhoun, of Newnan, Georgia, died August 1st, in the eighty-eighth year of his age. He was one of the oldest physicians in the State, though he had retired from practice about a dozen years ago. He graduated at the Charleston Medical College in 1831 and located in Newnan the following year where he has since resided. Dr. Calhoun was a successful practitioner and had accumulated large property. He was the father of Dr. A. W. Calhoun of this city.

Mr. John S. Prather, of Atlanta, was one hundred years old April 24th last, and is still in very fair health. The secret of his longevity, he says, has been regular habits, light eating and avoiding of medicines. Calomel has been his especial abhorrence. It has killed more people, he says, than the Napoleonic wars ever did. Above all things he advises moderate eating. He says gormandizers always die young, that is, *under seventy*. Mr. Prather hasn't "taken a dram" in sixty years, but has chewed tobacco for eightyfive years. Though not particularly related to his longevity, we may add that he has always voted the Democratic ticket and since the second election of James Monroe (his first vote), he has voted in every presidential election.

---

The secretary of the Birmingham (Alabama) Medical College has sent out the following circular letter to physicians:

"DEAR SIR:—Please send me on the enclosed postal card the names and addresses of any medical student or students you may know, or young men who expect to study medicine.

"For every student you may influence to attend our school the Faculty will give you $10. The said student or students to pay full tuition fees."

The *Journal of the American Medical Association* calls this an "Undignified Circular," which we think is a very mild characterization.

---

The Mississippi Valley Medical Association will meet at Louisville, October 5, 6, 7, 8, 1897. The Executive Committee met recently at Louisville, in conjunction with the local Committee of Arrangements, the following being present: Drs. Stucky, Grant, Mathews, Love, Holloway and Reynolds. It was determined to make the coming meeting the largest and best in the history of the Association, and everything points to a fulfillment of this endeavor. The railroads will make a round-trip rate of one and a third fare, or probably one fare. The address on surgery will be delivered by Dr. J. B. Murphy, Chicago; the address on medicine by Dr. John V. Shoemaker, Philadelphia. Title of papers should be sent to Dr. H. W. Loeb, Secretary, St. Louis, Mo.

---

The *American Journal of Surgery and Gynecology* (St. Louis) for June is a red-hot number. For several months the editor, Dr. Lanphear, has been publishing some spicy news and comments upon the "50-cent hospitals" and "Hospital Ticket Associations" which have infested that city, and his revelations, if true (and we have nowhere seen them denied), indicate a condition of affairs in medical St. Louis which might well make the shades of some of her departed great and good physicians weep. Passing by some of the smaller and personal exposures in this June number, we come to large scare heads which tell of *"Hospital and Dispensary Abuse in St. Louis. Runners Drum up Business for Prominent Men, Robbing the Doctors of the Country as well as the City. Nearly 400 Physicians of St. Louis are said to be Practically Starving."*

It appears that for a long while things have been getting so rotten in St. Louis, medically speaking, that a committee was appointed by the St. Louis Medical Society to make an exploratory investigation, so to say. In due time this committee reported that the medical colleges had been the chief malefactors, naming the Missouri Medical College as the "greatest offender against the well-being of the medical profession in particular and the community in general, in treating free patients well able to pay, thereby encouraging pauperism, not only of the laity, but of the doctor." Some hospitals and sanatoriums also come in for their share of condemnation. One of these sanatoriums established a "free dispensary in the heart of the most wealthy section of the city—not a pauper within a mile in any direction." It had "Free" printed on signs and cards, and "their agents or runners canvassed the country, distributing the cards or posters with the names of forty-five of the best doctors in the city."

Dr. Lanphear says that "this deplorable state of affairs is due to the contemptible methods of some of the most prominent teachers of medicine in the Mississippi valley." We trust that these delicate announcements will lead to a complete correction of all abuses, that the *entente cordiale* may be revived among our warring St. Louis brethren, and that medical matters out there will soon simmer down to a more harmonious and ethical basis.

